# Migration of South African health workers: the extent to which financial considerations influence internal flows and external movements

**DOI:** 10.1186/1472-6963-13-297

**Published:** 2013-08-06

**Authors:** Gavin George, Millicent Atujuna, Jeff Gow

**Affiliations:** 1 Health Economics and HIV and AIDS Research Division (HEARD), University of KwaZulu-Natal, Durban 4041, South Africa; 2 School of Commerce, University of Southern Queensland, Toowoomba, Australia

**Keywords:** Health workers, Public health sector, South Africa, Migration, Occupational specific dispensation

## Abstract

**Background:**

The loss of human resource capacity has had a severe impact on the health system in South Africa. This study investigates the causes of migration focussing on the role of salaries and benefits. Health professionals from public, private and non-governmental (NGO) health facilities located in selected peri–urban and urban areas in KwaZulu-Natal, South Africa were surveyed about their current positions and attitudes toward migration.

**Methods:**

The study uses cross-sectional data collected in 2009. A total of 694 health professionals (430 in the public sector, 133 in the NGO sector and 131 in the private sector) were surveyed. An additional 11 health professionals were purposively selected for in-depth interviews. Odds ratios with 95% confidence intervals were calculated to determine whether salaries influenced HWs decisions to migrate.

**Results:**

HWs decision to move was not positively associated with lower salaries. It was found, instead, that the consideration to move was determined by other factors including age, levels of stress experienced and the extent to which they were satisfied at their current place of work.

**Conclusions:**

The OSD appears to have lowered the risk of HWs migrating due to low salaries. However, the results also indicate that the South African Department of Health needs to improve working conditions for HWs within the public health sector to assist in retention.

## Background

The movement of health workers (HWs) both within and outside of South Africa has been recognised as a major problem for the health sector [1]. This phenomenon, it is argued, is increasing the strain on the already burdened public health sector, precisely because it leads to a shortage of skills and subsequent loss of capacity for health systems to deliver adequate health care [2,3]. The loss of HWs resulting from both internal and external movements places further pressure on remaining HWs by increasing their workload [4,5]. The South African case is not an isolated one. Many countries in sub-Saharan Africa (SSA) also face severe shortages of HWs precisely because HWs choose to move to better resourced countries [6]. The crisis is compounded by the fact that SSA countries have 25% of the world’s disease burden, and yet, it possesses only 1.3% of trained human resources for health (HRH) [6]. In South Africa, as a result of the movement of HWs, the doctor to population ratio was as low as 55 per 100,000 by 2010, whilst the nurse to population ratio had increased from 107 in 2003 to 383 per 100,000 [7,8]. These data however masks the unequal distribution found between the private and public sectors and between urban and rural settings [8].

Several studies examining the movement of HWs highlight the impact that migration has on health systems in developing countries [9-12]. These studies, regional in nature, reveal common ‘push’ and ‘pull’ factors including: lack of established posts and career opportunities; lack of availability of training at advanced levels; poor provision of service benefits; high rates of crime and lack of a secure work environment. All of these factors influence HWs’ decisions to migrate [13-15]. However, differences in potential earnings between source and destination are often posited as the key factor influencing the decision to move [3,15-21]. This wage gap between earnings of HWs in developed and developing countries is significant [22]. For example, an experienced theatre or intensive care unit nurse in South Africa earned about $9000 per year gross in 2005, whilst in Saudi Arabia the same nurse earned between $22,800 and $36,000 per year, tax free [23]. The variation is even greater when we consider the purchasing power parity across different countries. Table 1 summarises the relative wage differences between various source and destination countries for HWs.

**Table 1:** Relative monthly wage levels between source and destination countries, 2004 in US dollars (Purchasing power parity)

	**USDPPP monthly wage**			
	**Nurse**		**Doctor**	
***Source Countries***				
Chad	$	425	$	1050
Cote d’Ivoire	$	530	$	774
Ghana	$	206	$	473
Lesotho			$	3379
Malawi	$	489	$	868
Mozambique	$	441	$	2826
Namibia			$	2503
Philippines	$	380		
Sierra Leone	$	175	$	228
**South Africa**	$	1486	$	2836
Sri Lanka	$	407	$	1329
Trinidad and Tobago	$	913	$	1514
Uganda	$	38	$	67
Zambia	$	106	$	425
***Destination Countries***				
USA	$	3056	$	10554
UK	$	2576	$	7676
France	$	2133	$	5120
**Canada**	$	2812	$	8472
Australia	$	2832	$	5438

Source: [23].

In South Africa, the movement of HWs occurs in three ways. There is movement from rural to urban areas, from public to private sectors and outmigration to predominantly wealthier countries including the United States, United Kingdom, Canada and Australia [5,15,20-25]. The international mobility of HWs is, of course a key concern but figures of how many individuals have emigrated are not always easily available, and when these have been presented, they are obtained from destination countries. To date, there is no information on the exact number of South African HWs working abroad. The 2004 Organisation for Economic Co-operation and Development (OECD) report is the only study presenting detailed data on the migration of HWs from South Africa [26]. An extract of some of the data is presented in Table 2 and this shows that over 23,000 HWs had left South Africa up to 2004. The data is broken down in recognition of the fact that doctors, nurses and other health professionals have different education pathways, scopes of practice, and hence, their mobility potential will vary widely. Nonetheless, these figures are alarming and the real number is likely to be higher considering that some HWs travel on temporary visas and only become permanent much later [14]. Other studies have oultined the migration of South African HWs in similar numbers which are consistent with the OECD data [24]. The general indication is that compared to other professions (Education, Engineering and Accounting), the proportion of HWs migrating to wealthier economies is much higher [24]. Africa Health Placements, a South African NGO, estimated that of the 2,400 South African medical doctor graduates in 2006 and 2007, 75% would work in the private sector with only 300 opting to work in the public health sector with as few as 70 choosing to work in rural health facilities [27]. While in the past, movement tended to be among white males, seeking further training in developed countries, the trend today appears to be changing to include young, recently trained and mainly black nurses [25].

**Table 2:** Destination countries for South African health workers

	**Medical doctors**	**Nurses&midwives**	**Other health professionals**	**Total (N)**
***Destination Countries***				
Australia	1114	1085	1297	**3496**
Canada	1345	330	685	**2360**
New Zealand	550	423	618	**1596**
UK	3625	2923	2451	**8999**
USA	2282	2083	2591	**6956**
**Total**	**8921**	**6844**	**7642**	**23407**

Source: [26].

Decisions to move are linked to a web of intricate factors that have characterised the country over time. Specifically, the past political dispensation of apartheid led to large inequalities in service delivery which resulted mainly from the unequal distribution of expenditure budgets that favoured urban (mainly white) health facilities over rural (mainly black) health facilities. The effect of this is visible in the continuing differences in the quality of working environments and access to technology between urban and rural areas. The movement of HWs from rural areas to urban is facilitated by these differences but may also be determined by the higher salaries HWs earn in urban health facilities compared to rural health facilities [5,26,28-34].

In 2007, the South African Department of Health implemented the Occupation Specific Dispensation (OSD) policy in the public sector [35]. Dispensations for specific occupational categories included unique salary structures for each identified occupation, centrally determined grades and job profiles and career progression opportunities [36]. The salary package included increases in the base salary, 13th cheque (bonus), pension fund, medical aid, scarce skills allowance, commuted overtime, and in some instances a rural allowance. The OSD was specifically developed to address the rural/urban divide. The OSD resulted in all HRH being re-graded according to their qualifications and years of experience and remunerated at higher levels [36]. It was agreed that nursing would be the first profession to benefit from the OSD. Application to medical officers, medical specialists, dentists, pharmacists, and emergency medical services (EMS) occurred in 2008. The existing ranges of medical cadres were collapsed into one salary package [37]. Once implemented in 2008/09, salary packages increased substantially across cadres [35]. An entry level Medical Officer’s salary package increased by 21%, whilst Medical Officers with over 10 years experience saw their packages increase by between 46% and 68%, with Medical Specialists with over 10 years experience received increases between 29% to 49% [38]. Entry-level salaries for professional nurses and nursing assistants increased by 24%, while entry-level salaries for enrolled nurses increased by 20% [38].

Given the improved salaries and benefits that arose from the implementation of the OSD policy, this paper specifically investigates whether financial factors – in particular, salaries – are the primary reasons for HWs’ motivation when considering moving from their current place of work. The purpose of this paper is to contribute to an enhanced understanding of the issues surrounding HWs internal (intra country) flows and external (out of country) movements.

## Methods

### Study design, setting, population and sample size

The study uses cross-sectional data collected in late 2009. The study was undertaken in KwaZulu-Natal (KZN), one of the nine provinces of South Africa, and the most affected by HIV/AIDS. Health workers from facilities located in urban (Durban) and peri-urban (Stanger) areas in KwaZulu-Natal were selected. The HWs came from public, private and non-governmental (NGO) health facilities. The sample was selected using a proportional sampling method, wherein the number of participants was proportional to the number of HWs working in selected facilities. The largest number of professionals was selected from public health facilities (N = 430), followed by the NGO (N = 133) and then the private health facilities (N = 131). Thus a total of 694 HWS were selected. An additional 11 health professionals were purposively selected for subsequent in-depth interviews to add to the depth of understanding from the survey results.

### Survey instrument

The survey instrument was based upon the Immpact Toolkit titled ‘Health Worker Incentives Survey (HWIS)’ from the University of Aberdeen [39]. The focus was on HWs salaries and included sections covering their background, type of cadre, salaries earned, per diems, allowances and other benefits as well as any ‘moonlighting’ activities undertaken. Questions on job and workplace perceptions as well as questions on whether they considered moving, and, their preferred destinations, were included. These questionnaires were self-administered to doctors, professional nurses, enrolled nurses and nursing assistants.

### Statistical analysis

The analysis focused on selected migration indicators; considering to move, and considering to move abroad. The bi-variate distributions of the outcomes of interest are first examined and then the results of multivariate tests for key outcomes are presented and discussed. The outcomes considering to move, and, considering to move abroad were operationalised as dichotomies. As such, logistic regression with odds ratios and their corresponding 95% confidence intervals (CIs) for these outcomes were used in the multivariate analysis. Besides the main predictor of interest, salaries, the multivariate tests included variables such as age, marital status, level of education, length of time in their current job, workload, stress levels and job satisfaction as control variables. In recognising that doctors, registered nurses, enrolled nurses and nursing assistants vary widely in their scope of practice and hence their migration potential may vary significantly, the different models also include job title as a control variable. Because some of the respondents were drawn from the same departments and therefore share some unobserved characteristics, we obtained conservative, robust standard error estimates (by using the Huber/White/Sandwich estimator) to reduce the bias that might result from observation clustering. The bi-variate analysis and the multivariate model estimations were calculated using STATA version 11.

### Research ethics

Ethical approval was granted by the University of KwaZulu-Natal (UKZN) Humanities Research Ethics Committee (Ethical Approval Number: HSS/0703/07) was obtained. All participants involved in the study were informed about the nature of the study and what their participation would entail. Electronic and hard copies of the data are stored under lock and key at the Health Economics and HIV/AIDS Research Division (HEARD) offices, and are only accessible to the researchers involved in the study who are bound by confidentiality agreements.

### Limitations of the study

This study used of a cross-sectional design that entailed the gathering of data at only one point in time from nurses in selected health facilities in one province of South Africa. The generalisation of these findings to other HWs across the public, private and NGO sectors is limited by the proportional sampling method used and by the fact that the HWs participating in this study do not necessary represent HWs across the country working in these sectors [40]. The study obtained data only from doctors in the public sector and not from the NGO or private sectors, thus limiting the ability to determine how salaries influence this cadre within these two sectors.

## Results

### Characteristics of the HWs

Overall, the HWs surveyed had a mean age of 37 years. These were predominantly female (89%), employed as professional or enrolled nurses or nursing assistants (95%) whilst only a small cohort of the sample were doctors (5%). A considerable proportion of HWs (40%) reported earning relatively low salaries, ranging between R2,000-R6,000 ($285-$856) per month. The South African Rand/US Dollar exchange rate at the time of data collection was seven Rands to the US Dollar.

Table 3 presents descriptive characteristics, divided across four distinct samples: public, private and NGO sectors and the total sample. The data reveals that a large proportion of HWs (77%) indicated that they were not satisfied with their current job and that the vast majority of those were considering moving from their current job (72% overall). Of those who considered moving, 59% were public sector HWs which was proportional to their sample size (61%). About two thirds of public HWs (67%) also felt that they had a heavier workload than their colleagues in the private and NGO health sectors. Only one third (32%) of the sample reported not experiencing any serious level of stress.

**Table 3:** Descriptive statistics of the surveyed health workers

**Variables**	**Public sector**	**Private**	**NGO**	**General sample (%)**
**Outcome Variables**				
Considering to Move	60.90	14.89	24.2	72.45
Considering to Move Abroad	59.04	16.87	24.1	28.92
**Key Predictor variables**				
R2000-6000 ($285-$856)	56.51	17.84	25.65	40.88
R6000-10000 ($856-$1428)	67.04	10.61	22.35	27.20
R10000-20000 ($1428-$2857)	68.85	19.67	11.48	27.81
R20000+ ($2857+)	96.30	0	3.7	4.11
Average Income (actual Rand amounts)	12766	8172	7397	9602
**Other Predictor Variables**				
**Professional Cadre**				
Medical Doctors	100	0	0	4.96
Professional Nurses	59.98	21.40	19.07	37.52
Enrolled Nurses	60.98	19.92	19.11	35.91
Nursing Assistants	60.14	15.54	24.32	21.61
**Social Economic Characteristics**				
Current Age (mean)	37.31	35.30	37.59	99.01
Currently Married	56.58	25.62	17.79	41.35
Female	60.26	20.19	19.55	89.32
(Lower Sec + upper and vocation)	55.89	25.48	13.02	68.35
**Work Experience and Perceptions**				
Average Hours of Work Per Week	40.90	43.70	37.80	98.80
Length of Time Worked in Premise	4.33	2.39	4.34	99.03
Has a Heavy Workload	67.45	12.55	20.00	74.61
Not Stressed at Work	57.08	24.00	18.87	32.67
No Job Satisfaction	65.96	13.74	20.3	77.92
**N**	430 (61.96)	131 (18.88)	133 (19.16)	**694**

Notes: cell indicates the proportion present (versus absent) of the selected variable.

### Salaries as the main determinant for the movement of health workers

As the main focus of this paper, our analysis of preliminary associations presented in Table 4 shows the bi-variate analysis of the distribution of salaries earned, with the subsample of HWs who were considering moving (72%). At this level of analysis, we found no indications that low salaries were correlated with HWs decisions to move.

**Table 4:** Preliminary association of the effect of salaries on health workers decisions to move (N = 694)

**Selected behavioural variables**					
	**Monthly salaries**	**Monthly salaries**	**Monthly salaries**	**Monthly salaries**	**Total (%)**
	**R20,000+**	**R2000-R6000**	**R6000-R10000**	**R10000-R20,000**	
	**($2857+)**	**($285 -$856)**	**($856-$1428)**	**($1428-$2857)**	
Considering to Move	3.25	40.77	26.98	29.01	72.45
	x2=7.03+				(694)
*Destinations*					
Move Abroad	2.44	37.80	26.03	32.92	29.71
	x2=3.37				(694)

Cells indicate the proportion present versus absence of the selected variables above.

Chi square significance levels ^+^p < 0.1, *p < 0.05, **p < 0.01, ***p < 0.001.

Total percent is a proportion of the N value underneath it.

Each dependent variable, i.e. considering to move, move abroad, private, NGO, and public health sectors is.

Presented with an associated chi square value and a corresponding level of significance.

The results of a multivariate logistic regression for the effect of salaries on HWs decision to move from their current work are presented in Table 5. Three Models were used for estimation purposes. The first Model (base Model) examined the effect of salaries without moderating for other variables. In this Model, the results which were moderate at the bi-variate level have become more pronounced. Earning lower salaries was not statistically significant and was not correlated with the decision of HWs to move from their current job. The second Model examined the effect of salaries controlling for the effect of socio-economic status (SES) characteristics. Even here, no significant results regarding the effect of salaries on HWs decision to move is observed. Instead, the results indicate that their decision to move from their current place of work was primarily influenced by their age. Accordingly, HWs were less likely to consider migrating as they got older (OR = 0.95, 95% CI: 0.92-0.97). In-depth interviews corroborated these findings by suggesting that older HWs were attracted by a sense of stability, and therefore would not consider migrating.

**Table 5:** Salaries and other factors predicting decisions to migrate (N = 694)

	**Model 1**		**Model 2**		**Model 3**	
	**OR**	**95%CI**	**OR**	**95%CI**	**OR**	**95%CI**
**Monthly Salaries**						
R20000+ ($2857)						
R2000-6000 ($285-$856)	1.51	0.37 - 6.16	1.91	0.31 - 1.71	2.25	0.23 - 2.51
R6000-10000 ($856-$1428)	1.79	0.46 - 6.91	2.68	0.45 - 5.06	3.95	0.49 - 3.67
R10000-20000 ($1428-$2857)	0.71	0.19 - 2.63	1.88	0.34 - 4.21	4.19+	0.53 - 3.78
**Social Economic Characteristics**						
***Job Title***						
(Medical Doctors)						
Professional Nurses			0.55	0.08 - 3.73	0.38	0.04 - 3.53
Enrolled Nurses			0.53	0.07 - 3.88	0.77	0.08 - 7.35
Nursing Assistants			0.67	0.07 - 5.63	1.39	0.11 - 7.16
Current Age			0.95***	0.92 - 0.97	0.93***	0.88 - 0.97
Currently Married			1.02	0.59 - 1.75	1.34	0.65 - 2.75
Female			0.65	0.23 - 1.98	1.27	0.35 - 4.53
(Lower Sec + upper and vocation)			1.21	0.62 - 2.38	1.36	0.57 - 3.23
**Work Experience and Perceptions**						
Average Hours of Work Per Week					0.99	0.95 - 1.03
Length of Time Worked					0.61	0.26 - 1.14
Does Not Have a Heavy Workload					1.11	0.92 - 1.34
Not Stressed at Work					0.46**	0.21 - 1.01
No Job Satisfaction					3.58***	1.72 - 4.54
Log Likelihood	−276.956		−168.221		−106.84	
Pseudo-R2	0.016		0.033		0.0085	
**N**	**487**		**318**		**239**	

Notes: Reference category in parenthesis.

Significant at + p < 0.1, *p < 0.05, **p < 0.01, ***p < 0.001.

OR: Odds ratios among lower and higher salary earners and other SES and work experience variables.

CI: Confidence interval at 95%.

Analysis controlling for both the SES characteristics and the work experience and perceptions of HWs is presented in Model three. When all these factors are controlled for, the results remain largely unchanged. Salaries still do not predict HWs decisions to move from their current place of work. We find, however, that in addition to age, the extent to which HWs were satisfied with their jobs (OR = 3.58, 95% CI: 1.72-4.54) and the level of stress experienced (OR = 0.46, 95% CI: 0.21-1.01) also determined their decisions to consider moving. These two results are very statistically significant. In short, HWs who were not satisfied at work were three times more likely to move than their counterparts who were happy in their current job. HWs who reported that they were not stressed at work were 54% less likely to consider migrating from their current place of work. When moving abroad is used as a dependent variable, the same factors observed earlier are found to determine HWs decision to consider moving, as presented in Table 6.

**Table 6:** Salaries and other factors predicting decisions to migrate abroad (N = 694)

	**Model 1**		**Model 2**		**Model 3**	
	**OR**	**95%CI**	**OR**	**95%CI**	**OR**	**95%CI**
**Monthly Salaries**						
R20000+ ($2857)						
R2000-6000 ($285-$856)	1.14	0.16 - 7.63	0.85	0.08 - 3.51	0.81	0.66 - 1.88
R6000-10000 ($856-$1428)	1.04	0.16 - 6.91	0.89	0.09 - 6.06	1.16	0.11 - 2.67
R10000-20000 ($1428-$2857)	1.62	0.15 - 2.02	1.24	0.15 - 1.06	1.48	0.14 - 5.78
**Social Economic Characteristics**						
**Job Title**						
(Medical Doctors)						
Professional Nurses			0.75	0.04 - 3.73	0.54	0.02 - 3.89
Enrolled Nurses			0.91	0.42 - 8.38	0.07	0.02 - 9.35
Nursing Assistants			0.81	0.03 - 9.63	0.65	0.01 - 2.16
Current Age			0.43**	0.98 - 1.86	1.04*	0.99 - 1.11
Currently Married			1.77	0.42 - 7.17	1.94	0.36 - 1.29
Female			1.44	0.71 - 5.98	1.29	0.57 - 2.93
(Lower Sec + upper and vocation)			2.46*	1.01 - 5.94	2.11*	0.76 - 5.23
**Work experience and perceptions**						
Average hours of work per week					1.03	0.95 - 1.03
Length of time worked					0.71	0.23 - 2.18
Does not have a heavy workload					0.89	0.71 - 1.12
Not stressed at work					0.95*	0.77 - 4.89
No Job Satisfaction					1.58*	1.24 - 1.87
Log likelihood	−163.502		−99.561		−80.189	
Pseudo-R^2^	0.0169		0.06		0.0095	
**N**	**275**		**182**		**154**	

Notes: Reference category in Parenthesis.

Significant at + P < 0.1, *p < 0.05, **p < 0.01, ***p < 0.001.

OR: Odds Ratios among lower and higher salary earners and other SES and work experience variables.

CI: Confidence interval at 95%.

## Discussion

Several studies in other countries have shown that economic factors are potentially the most significant reason as to why a large number of HWs move from one health sector to another, and are the most determinant factor explaining the movement of HWs to developed countries. In South Africa, economic reasons – salaries in particular – appear to have been one of the major reasons why HWs were migrating, particularly in earlier years (2002–2006) when major destination countries actively sought to recruit South Africa HWs. In an attempt to reduce these losses the South African Department of Health implemented the OSD policy in order to correct the large disparities in salary earnings between the private and public sector and between source (South Africa) and destination countries. Although the gap in salary earnings still exists in certain instances, it has been narrowed down by a considerable degree [41]. As the results presented here indicate, HWs decisions to migrate are not linked to the salaries they earn in their current jobs. Instead, HWs decision to consider moving is largely influenced by their age, their level of stress at work and the extent to which they feel happy in their current job. Public sector HWs are particularly susceptible to migration due a number of challenges which this and other studies have found [40]. Reasons for the desire to move out of the public sector and into the private sector or abroad revolve around both push and pull factors. The former includes those factors that make other settings more attractive than their current job, such as higher wages, better working conditions and better opportunities for professional advancement in foreign countries. The latter comprises factors which drive staff out of the public sector. For instance, lack of management support, lack of supervision, poor working conditions, work overload, emotional burn out, lack of appropriate resources and poor infrastructure [42]. Much of the challenges faced by the public health care system are as a result of inadequate numbers of HWs. Overall, there is a shortage of 80,000 HWs in the public sector. Approximately 30% of medical practitioners, 60% of nurses and 15% of pharmacists are employed in the public sector, yet they serve approximately 85% of South Africa’s population. Current production rates of HWs have not kept abreast of population growth rates and, without significant improvement, will not be able to offset the shortfall of HWs created by migration [37].

The results of this study resonate well with the long standing behavioural theories developed by Maslow and Herzberg which suggest that the movement of labour should be understood within the complex processes which shape aspects such as job satisfaction and several other aspects that affect an individual worker [43,44]. Such processes include the extent to which work environments make for happier workers and are at the same time providing adequate motivation for workers. This is not the case in most developing countries where health facilities in general are characterised by poor infrastructure, management problems, unequal distribution of resources and high numbers of people seeking treatment, and in the case of South Africa accentuated by the high prevalence of HIV/AIDS in the population. Combined, these problems are likely to have an adverse effect on HWs’ motivation levels and ultimately the ability to perform their work.

## Conclusions

Despite public perceptions, the extent to which remuneration is the key motivating factor behind HWs movement within and out of South Africa has been overestimated. HWs decisions to move appear to be motivated by a mixture of factors including high levels of stress as a result of increased workloads and other challenges faced in their current job. The movement of HWs previously linked in the literature with lower salaries, appears to have been minimised by the implementation of the OSD policy by the South African Department of Health [40,41]. However, the most pressing need is to rectify the mal-distribution of HWs between the public and private sectors and rural and urban areas. Thus, strategies aimed at ameliorating the impact of HW shortages should concentrate on the retention of HWs being produced whilst ensuring that working within the public sector is appealing to prospective employees. Improved salaries through the OSD have improved the economic position of public sector HWs yet non-financial challenges plaguing the sector are bigger deterrents to current and prospective HWs. The challenge for the South African Department of Health is improve the public health care system in order to enable HWs to perform their duties in suitable environments, to keep them motivated and intent on staying.

## Abbreviations

CIs: Confidence intervals; HEARD: Health economics and HIV and aids research division; UKZN: University of KwaZulu-Natal; HWs: Health workers; OSD: Occupational specific dispensation; OECD: Organisation for economic co-operation and development; KZN: KwaZulu-Natal; NGO: Non-governmental organisation; SSA: Sub-Saharan Africa; HRH: Human resources for health.

## Competing interests

The authors declared that they have no competing interests.

## Author’s contributions

GG designed the study, interpreted the data and prepared the manuscript. MA conducted the analysis and contributed to the writing of the manuscript. JG interpreted the data and assisted in preparing the manuscript. All authors read and approved the final manuscript.

## Author’s information

GG is a Senior Researcher based at HEARD, UKZN, South Africa. MA is a former Junior Researcher at HEARD. JG is a Professor of Economics and the Economics and Politics Discipline Leader in the School of Commerce, University of Southern Queensland, Toowoomba, Australia. JG is also a HEARD Research Associate.

## Pre-publication history

The pre-publication history for this paper can be accessed here:

http://www.biomedcentral.com/1472-6963/13/297/prepub
